# Supplementary material for: Are carbon emissions trading and green financial instruments synergistic? -Comprehensive quantitative research based on content analysis
Source: PLoS One. 2024 Mar 7;19(3):e0298601. doi: 10.1371/journal.pone.0298601 (PMC10919601; doi:10.1371/journal.pone.0298601)
Supplement: S1 Data — (ZIP) [file pone.0298601.s001.zip › Data/地级市Z_F工作报告.zip!/─┌├╔╣┼/╢⌡╢√╢α╦╣╩╨/└┤╫╘╣1⁄2╓┌║┼í╛┬φ┐╦╩2╛▌═°í┐_2017.docx]

各位代表：

现在，我代表市人民政府向大会报告工作，请予审议，并请市政协委员和其他列席人员提出意见。

一、2016年工作回顾

过去一年，是我市经受考验、逆势奋进、取得丰硕成果的一年。一年来，全市上下认真学习贯彻习近平总书记系列重要讲话和考察内蒙古重要讲话精神，深入落实中央、自治区和市委重大决策部署，积极应对挑战，奋力攻坚克难，较好地完成了各项目标任务，经济社会发展取得新成效，实现“十三五”良好开局。

——整体经济稳中向好。完成地区生产总值4417.9亿元，增长7.3%，增速高于全国、全区平均水平；公共财政预算收入451亿元，同口径增长3.8%；固定资产投资3050亿元，增长12%；几项主要经济指标继续保持全区第一，实现了高基数上的平稳较快增长。

——发展后劲显著增强。蒙西至天津南、上海庙至山东两条特高压电力通道顺利推进，京东方二期等项目即将建成，中煤烯烃、伊泰精细化学品、中天合创煤炭深加工等26个项目正式投产，一批构筑多年、投资巨大、引领转型升级的重大项目取得突破性进展，为全市产业发展奠定了更加坚实基础。

——现代服务业蓬勃发展。响沙湾莲花酒店、万家惠欢乐世界等一批特色文旅项目投入运营，国际创意文化大会、美丽乡村旅游节等一批品牌节庆活动成功举办，全市旅游人数首次突破千万人次，被列为国家全域旅游示范区创建单位。天津港、天津物产等国内大型物流企业入驻，电子商务交易量成倍增长，物流业发展势头迅猛。

——发展环境全面改善。康巴什区正式经国务院批准设立。呼包鄂动车开通运行，鄂尔多斯机场升级为国际机场，国际航空口岸正式对外开放，综合保税区申报取得重大进展，大开放格局初步形成。“十个全覆盖”任务基本完成，脱贫攻坚取得决定性胜利，农村牧区面貌焕然一新，获评中国美丽乡村建设示范地区。

——社会大局和谐稳定。坚持将80%以上的可用财力用于民生保障，重点民生工程全面完成。解决了一批社会保障、环境保护、食药安全等涉及群众切身利益的突出问题。城乡常住居民人均可支配收入分别增长7.4%和7.3%，达到40221元和15480元，人民群众生活更加殷实。

一年来，我们主要做了以下工作：

（一）全力保持经济平稳运行。集中精力抓项目扩投资，组织实施亿元以上重大项目210项。创新投融资方式，对接设立产业基金15支，总规模近500亿元，新增社会融资703.8亿元。用改革的办法破解要素制约，置换黄河用水指标1.25亿立方米；59个项目开展排污权交易；17家企业参加京蒙跨区域碳排放权交易。加大招商引资力度，引进项目200项，总投资590亿元；实际利用外资19.5亿美元，总量居全区第一。努力扩大消费和贸易，社会消费品零售总额增长10%；进出口贸易额增长80%，成为自治区唯一的全国外贸百强城市。

（二）加快供给侧结构性改革。完成30万吨煤炭、108万吨水泥、25万吨电石产能退出任务。大力推进棚户区改造，化解存量商品房589.8万平方米，被住建部确定为房地产去库存试点城市。积极稳妥去杠杆，企业直接融资达到151.1亿元。出台了支持工商企业降本增效一系列政策措施，累计降低企业用电成本14.7亿元，减免税费59.8亿元，落实各类补贴11.3亿元，规模以上工业企业利润增长29.3%。优化煤炭供给结构，成功注册“鄂尔多斯煤”商标，全年销售煤炭5.4亿吨。实施补短板系列工程，基础设施、科技创新等地区发展的薄弱环节明显增强。

（三）积极推进产业转型升级。持续优化工业结构。新增电力装机164万千瓦，煤化工产能540万吨，煤炭就地转化率提高1个百分点。大力发展装备制造、电子信息等战略性新兴产业，非煤产业增加值占到规模以上工业增加值的48.3%。加快发展现代服务业。旅游人数和收入分别增长19.9%和22.7%，成为国内旅游度假和休闲养生热点地区。存款余额首次突破3000亿元。我市被确定为国家第一批医养结合试点城市。稳步发展现代农牧业，粮食产量、牧业年度牲畜存栏量实现“十二连增”，农牧民与龙头企业利益联结更加紧密。

（四）持续加大改革创新力度。在全区率先编制并公布权责清单，率先推行个体工商户“两证合一”和企业“五证合一、一照一码”登记制度改革，全面实行“单一窗口”受理模式，企业和群众办事时间缩短80%以上，市场主体注册资本增长1.5倍。建成了覆盖市、旗、乡、村四级的政务服务网络，获评全国优秀政务服务平台。不动产统一登记制度正式施行。国有企业整合重组全面完成。出台了促进科技创新30条政策措施，建成院士专家工作站2个、技术转移中心3个、自治区级以上众创空间15家，新增国家高新技术企业13家，申请专利数增长84.7%，大众创业、万众创新蓬勃展开。

（五）推动城乡区域协调发展。中心城区一体化步伐加快。率先在公共交通、电力保障等方面实现一体化运营。新建改造市政管网186.1公里、城镇道路102.9公里，新增城区绿地575万平方米。国家智慧城市、信息消费试点城市、“宽带中国”示范城市建设扎实推进，进入全国智慧城市建设50强。深化户籍制度改革，常住人口城镇化率达73%以上。全面改善农村牧区环境，735个行政村和较大自然村实现基本公共服务全覆盖，健全后续管护长效机制，建成自治区级美丽宜居示范镇村8个，乌审旗无定河镇获评“中国最美乡镇”。加快基础设施建设，新增高等级公路186公里、农村牧区公路3570公里、铁路运营里程114公里。

（六）全面提升绿色发展水平。加大生态建设力度，完成林业建设145万亩、草原建设385万亩，治理水土流失180.5万亩，森林覆盖率提高0.2个百分点。深入开展国家环保模范城市创建，新标准下空气质量优良天数达到317天。全面完成年度节能减排任务。强化环保执法能力建设，建立污染防治区域部门联动机制，在全区率先建立网格化管理平台，环保重点企业实现在线监控全覆盖。推进生态文明体制改革，自然资源资产负债表编制、领导干部自然资源资产离任审计试点进展顺利，《鄂尔多斯市环境保护条例》正式出台。

（七）稳步提高民生保障能力。新增城镇就业2.86万人，城镇登记失业率控制在2.88%较低水平。回购各类保障性住房15392套。企业职工基础养老金人均提高167元，城镇居民医保和新农合筹资标准每人每月分别提高64.4元和66元。更加关注特殊困难群体利益，在全区率先施行困难残疾人生活补贴和重度残疾人护理补贴制度。加快社会事业发展。新建、续建中小学幼儿园55所，学前教育入园率提高到96%。高考普通类本科上线率达到69.4%，位居全区第二。公立医院改革向纵深推进，在全区率先推行医疗服务价格和药品招标采购机制改革。两孩政策全面实施。“十个一”文化工程扎实推进，被文化部列为全国首批文化消费试点城市。

（八）全力防范化解风险隐患。出台防控地方金融风险工作方案，帮扶95家重点困难企业化解兑付担保风险，企业转贷续贷306.6亿元，银行不良贷款余额、不良率实现“双下降”，守住了不发生区域性金融风险的底线。加快金融纠纷案件处置，有力保障了群众合法利益。高度重视政府债务风险，争取政府债券置换资金641.8亿元，增长71.2%。持续开展安全生产大检查，安全形势总体平稳。建成市、旗两级食品药品综合监管平台，保障了人民群众“舌尖上的安全”。加大矛盾排查化解力度，一批信访积案得到有效解决。刑事和治安案件下降16.8%和3.7%，社会治安形势持续向好。

（九）不断加强政府自身建设。坚持依法行政，自觉接受市人大及其常委会的法律监督和工作监督，定期向人大报告工作，提请人大审议制定地方性法规2部。自觉接受市政协民主监督，主动听取各方面意见建议。人大代表建议和政协委员提案全部办结。严格执行“三重一大”集体决策制度，法律顾问制度全面推行。扎实开展“两学一做”学习教育，严格执行作风建设有关规定，政府系统作风明显改善。狠抓决策执行和工作落实，出台了提高政府执行力的实施意见，行政效能和服务水平不断提升。严控行政支出，党政机关和参公事业单位公车改革全面完成，“三公”经费下降7.6%。加强审计监督和行政监察，廉政建设和反腐败斗争取得积极成效。

此外，精神文明和民主法治建设全面加强，国防动员、人民防空、民族宗教、新闻广电、外事侨务、史志档案、气象地震、妇女儿童、老龄事业等各项工作均取得新进展。

各位代表，回首过去的一年，困难前所未有，成绩弥足珍贵。这是中央、自治区坚强领导、关怀支持的结果，是市委总揽全局、科学决策的结果，是全市各族干部群众同心同德、奋力拼搏的结果。在此，我代表市人民政府，向全市各族人民和所有关心、支持、参与鄂尔多斯建设发展的社会各界人士，表示衷心的感谢，并致以崇高的敬意！

在肯定成绩的同时，我们也清醒地认识到，当前我市发展中还面临一些突出的问题和挑战。主要是：产业规模还不大，层次低、链条短，竞争力不够强，转型升级任务艰巨；科技创新能力弱，对外开放程度低，资源环境和要素制约进一步加大，经济增长内生动力不足；城乡发展不够协调，农村牧区相对落后，城市治理水平仍有待提升；环境保护、安全生产、信访维稳等领域风险隐患还很大，一些关系群众切身利益的问题还亟待解决；政府自身建设和干部作风转变与人民群众期盼还有一定差距。对此，我们将采取有力措施，认真加以解决。

二、2017年工作安排

今年，是实施“十三五”规划的重要一年，是供给侧结构性改革的深化之年。我们面临的形势依然错综复杂，有利条件和不利因素并存。新的形势，需要我们自加压力，主动担当，以改革创新的精神和一往无前的勇气，响应习近平总书记号召，撸起袖子加油干，落实自治区党委、政府要求，跳起来摘桃子，抓住重点，攻克难点，聚焦热点，打造亮点，以新的思路、方法和举措，不断解决前进道路上的新矛盾、新问题，积极进取，扎实工作，确保圆满完成全年各项目标任务。

今年政府工作的总体思路是：全面贯彻党的十八大和十八届三中、四中、五中、六中全会精神，紧紧围绕自治区第十次党代会、自治区党委十届二次全会和市第四次党代会、市委四届三次全会决策部署，统筹推进“五位一体”总体布局，协调推进“四个全面”战略布局，坚持稳中求进工作总基调，牢固树立和贯彻落实新发展理念，适应把握引领经济发展新常态，以提高发展质量和效益为中心，以推进供给侧结构性改革为主线，坚决守住发展、生态、民生底线，振兴实体经济，深化转型发展，大力促进“五化协同”，全力做好稳增长、促改革、调结构、惠民生、防风险各项工作，全面推动经济社会发展再上新水平，以优异成绩迎接自治区成立70周年和党的十九大胜利召开。

主要预期目标是：地区生产总值增长7.5%左右，规模以上工业增加值增长8.5%左右，固定资产投资增长12%左右，公共财政预算收入同口径增长6%左右，社会消费品零售总额增长10%左右，城乡常住居民人均可支配收入分别增长8%和8.5%左右。

今年，市政府将重点做好八个方面工作：

（一）坚持供需两侧发力，引领经济发展新常态。

持续扩大有效投资。把项目建设作为重中之重，组织实施亿元以上重点项目304项，形成新的项目建设热潮。加大项目前期工作力度，力争纳林河矿区、准格尔中部矿区等重大规划获批，北控煤制气等项目核准。建立重大项目动态储备、调整机制，制定项目滚动投资计划，确保项目建设连续性。创新招商引资方式，通过产业链招商、平台招商、以商招商等方式，在煤化工产业延伸、高新技术产业、产学研一体、现代服务业等领域再引进实施一批重大项目，力争引资规模达到600亿元以上。拓宽投融资渠道，支持企业上市融资，新增社会融资750亿元以上；继续做大产业基金规模，为项目建设提供资金保障。

不断深化供给侧结构性改革。坚定不移推进“三去一降一补”，切实提高经济发展活力。去产能方面，依法依规退出不合规、不达标产能，严格控制新增过剩产能。继续推进煤炭企业兼并重组，引导退出煤炭落后产能90万吨。优化煤炭产品供给，分级分质对接市场需求，原煤洗选率提高到90%以上，进一步扩大我市优质煤炭市场占有率。去库存方面，把去库存与推进城镇化结合起来，统筹解决好房屋产权登记、项目重组、兑换凭证互通互用等问题，积极拓宽融资渠道，扩大棚改货币化安置比例，实施棚户区改造1.6万户，再消化房地产库存500万平方米左右，引导我市房地产市场回归健康发展轨道。去杠杆方面，继续强化政府、银行和企业之间的合作，突出抓好金融风险防范，设立地方金融资产管理公司，开展风险企业帮扶和不良贷款化解专项行动，维护好广大企业和金融机构的合法权益。严格规范政府举债行为，坚决不允许出现政府贷款违约现象，确保不发生系统性、区域性金融风险。降成本方面，打好政策“组合拳”，进一步降低企业税费负担和融资、人工、物流、用地、用能成本。引导企业通过强化管理和创新，降低成本、提高效益。补短板方面，围绕科技创新、公共服务、民生改善等领域，组织实施补短板专项工程，全面提高发展的协调性和平衡性。

积极释放消费潜力。加快综合保税区、内陆港和跨境电子商务基础设施建设，建成运营一批进口商品体验馆。推动北京动物园服装批发市场入驻，确保康巴什康镇、东胜铜川二手车交易市场等重点项目建成投用，培育新的消费热点。办好绒博会、煤博会等活动，聚集人气商气，促进节庆会展消费。加强苏木乡镇物流配送网点建设，挖掘农村牧区消费潜力。发扬工匠精神，打造百年老店，开展质量提升行动和“商标品牌培育年”活动。加快创建放心消费城市，严格市场监管和质量监管，严惩商业欺诈、制假售假等行为，全面改善地区消费环境。

大力发展非公经济。把非公经济发展摆在更加重要的位置，认真落实促进非公经济发展的各项优惠政策，建立行业准入负面清单，鼓励非公经济通过PPP等模式依法平等进入清单之外的领域。全力扶持中小微企业发展，构建大企业顶天立地、中小企业铺天盖地的发展格局。保护好企业家精神，进一步营造亲商、敬商、安商、富商的良好社会氛围，让民企在地区发展大潮中扛大梁、挑重担、作出更大贡献。

（二）不断优化产业结构，实现转型发展新突破。

促进传统产业提质增效。推动煤矿绿色生产，推广安全高效开采方式，煤矿采区回采率提高到80%以上，机械化开采程度提高到95%以上。加快清洁能源电力外送基地建设，抓好山东能源盛鲁电厂、国电双维电厂等电源点项目，确保蒙西至天津南、上海庙至山东特高压电力通道建成。加快煤电铝一体化，积极推进鑫恒铝业产能置换，开工建设蒙泰一期45万吨铝板带项目。大力发展精细化工，推动伊泰200万吨煤制油、神华煤制油二三线、国电投烯烃等项目建设，确保中天合创煤炭深加工、伊泰精细化学品、新能稳定轻烃等项目达产达效。稳步推进煤层气、页岩气勘探开发，常规天然气产能保持在300亿立方米左右。持续推进羊绒产业振兴计划，依靠科技创新提升陶瓷、冶金、建材等传统优势产业发展水平。

促进新兴产业做大做强。提升装备制造业发展层次。积极申报“中国制造2025”试点示范城市。加强与以色列集团等大型企业合作，引进新能源汽车、智能机器人等项目。加快国际航空口岸建设，培育发展航空器生产和航空培训、航空物流等新型业态。加快培育发展云计算、大数据产业，把我市建成自治区大数据产业发展的重要增长极。加大新能源开发力度，争取达拉特旗库布其沙漠光伏发电基地启动建设。积极引进培育石墨烯、高纯氧化铝、碳纤维等新材料产业。力争非煤产业增加值占比再提高5个百分点左右。

促进现代服务业融合配套。实施全域旅游发展战略，高起点编制全域旅游发展规划和品牌线路、自驾车营地、公共服务体系建设等专项规划，加快国家旅游改革先行示范区建设，举办好鄂尔多斯旅游推介会暨世界旅游城市形象大使选拔赛、冰雪旅游节、库布其沙漠主题和红色旅游主题等重大活动，加紧实施黄河大峡谷、蒙古源流文化产业园等重点项目，集中打造10个乡村旅游示范村和50个乡村旅游特色农牧家乐，继续加强旅游基础设施建设，拓展旅游市场，提升鄂尔多斯旅游品牌影响力。年内旅游接待人数和旅游收入分别增长18%和15%以上。大力发展现代物流业，深化与天津物产、天津港等大型物流企业合作，集中推进空港、棋盘井等现代物流集聚区建设。发展文化创意产业，推进国家文化消费试点城市建设，办好国际创意文化大会、文博会等活动。发展壮大金融业，大力引进金融机构，积极筹建民营银行，深化农信社改革，推动市内大企业集团发起设立消费金融公司、融资租赁公司等新型金融机构。拓展健康养老产业发展内涵，加快仕博国际健康城等项目建设，打造健康产业集约发展示范区。

促进现代农牧业转型升级。优化农牧业产业布局，在两河流域、西部牧区、城郊区分别集中发展种养结合大农业、现代草原畜牧业和休闲观光农业。强化耕地保护和质量提升，发展适度规模经营，年内流转土地10万亩、草牧场160万亩以上，建成公司化养殖园区10处、养殖大户300家、家庭农牧场100家。推进农牧业供给侧结构性改革，深入落实“粮改饲”及“稳羊增牛”战略，新打造100个产业结构调整及产业发展典型示范村。促进农牧业品牌化发展，建立农牧业标准化体系，建成市级农畜产品质量安全检测中心。推进农畜产品精深加工，培育10个年产值超亿元的农牧业产业链条，力争加工转化率达到60%以上。完善农畜产品流通体系，新建网上农贸市场10家、冷链流通仓库5个、专业合作社100家，带动农牧户增收致富。

（三）全面深化改革创新，培育支撑发展新动能。

加快推进基础性关键性改革。突出抓好总书记指示自治区先行先试的三项重点改革任务，确保在建立生态环境损害责任终身追究制度、提高农牧民产业化经营收益率、扩大与俄蒙合作交流等方面取得新突破。继续深化“放管服”改革，完善权责清单，全面推行“双随机、一公开”监管制度。进一步简化审批程序，做到审批信息互联互通、服务事项网上运行，真正实现“推开一扇门，办成一揽子事”。深化商事制度改革，实现工商登记全程电子化和简易注销登记。持续推进电力多边交易、碳排放权交易、水权交易等改革，破解发展要素制约。深化农村牧区综合改革，推动土地草牧场“三权分置”和国有林场改革，完成农村牧区土地草牧场确权登记颁证任务。

深入实施创新驱动发展战略。深化科技体制改革，设立科技创新基金，营造有利于创新驱动发展的市场和社会环境。加快市高新区发展步伐，吸引高端研发机构和高新技术企业入驻。健全产学研协同创新机制，积极推动与北京协同创新研究院、中科院深圳先进技术研究院等合作设立创新中心。实施高新技术企业培育行动计划，新增高新技术企业8家、自治区级研发机构3家以上。强化重大关键技术攻关，着力攻克不同煤种气化、煤炭分级利用、高盐废水处理等共性关键技术难题。组建全市众创空间联盟，推动各类科研平台向社会开放，共享创新创业资源，建设创新之城、创业之城。加大各类人才培养引进力度，满足地区发展需求。

全方位扩大对外开放。主动对接、全面融入呼包鄂协同发展战略，实现优势互补、协同共进。加强产业协作，联合呼包二市申建内陆自贸区，合作共建西部装备制造基地、国家大数据综合试验区。加快基础设施互联互通，打造呼包鄂中心城区1小时快速铁路圈和2小时公路圈。加强生态共建，建立环境污染防治联防联控机制。推进就业、教育、医疗等公共服务共建共享。加强与“一带一路”国家和京津冀、长江经济带、珠三角、晋陕宁蒙等区域协作，主动参与呼包银榆经济区建设，积极承接发达地区高端、环保产业转移，提升区域合作水平。

（四）全面改善基础条件，构建协调发展新格局。

推进以人为核心的新型城镇化。加快中心城区一体化进程。编制并实施中心城区道路、绿化、供水等一体化规划。推进东胜至机场轻轨、东胜吉劳庆川和康巴什环城水系景观等项目建设。巩固全国文明城市创建成果，提高城市精细化管理水平，出台城市绿化、物业管理条例，深入开展住宅小区文明创建和城市精细化治理行动。大力发展县域经济，加快县域城镇化进程，开展“生态宜居美丽县城”创建行动，培育一批国家和自治区特色小镇。实施美丽乡村三年行动计划，推进美丽乡村建设与产业发展深度融合。推动城市优质公共服务向基层延伸，改造农村牧区危房3000户以上，在全市150个行政村开展垃圾、污水集中收集和治理试点，全面改善农村牧区生产生活条件，努力让农牧民共享城市公共服务。

推进基础设施网络互联互通。重点抓好“七网”建设。铁路网方面，全力推进包西铁路客运专线包榆段前期工作，力争“十三五”开工建设。加快蒙华、鄂前旗至上海庙铁路等项目建设进度，确保沿河、准朔铁路等项目完工，呼准鄂改客专项目建成，新增铁路运营里程200公里。公路网方面，启动实施包茂高速包头至东胜改扩建工程、呼市至鄂尔多斯高速等项目，加快通史至靖边、龙口至大饭铺、敖镇至东道梁等出省通道建设，确保沿黄公路一级改高速、察汗淖至棋盘井一级改高速等项目建成通车。新增高速公路260公里，改建农村牧区公路500公里。航空网方面，优化航线网络布局和运力结构，增加通航城市和国际航线。启动建设鄂前旗、杭锦旗2个通用机场。市政网方面，加大对城市老旧管网改造力度，制定地下综合管廊和海绵城市建设方案，在中心城区率先试点。水利网方面，实施黄河内蒙古段二期防洪工程、西北节水增效项目等重点水利工程，完成盟市水权转让一期和拦沙换水试点工程建设，增强区域水资源保障能力。能源网方面，加快陕京四线天然气管输工程建设，力争鄂安沧输气管道、蒙西煤制气外输管道获批开工。全面实施“气化鄂尔多斯”等工程，提高农村牧区清洁能源普及率。通信网方面，加强信息基础设施建设，确保鄂尔多斯至北京直通光缆建成运行。创新机制、整合资源，加大信息化平台建设力度，基本完成“智慧鄂尔多斯”、“宽带中国”示范城市建设任务。

（五）秉持绿色发展理念，开创生态文明新局面。

更大力度保护生态。实施好京津风沙源治理、天然林保护等重点生态工程，突出抓好沿黄河绿化风光带、环城区城镇绿化带、沿沙漠沙地绿化带和沿路沿线绿化带“四带工程”，完成林业建设120万亩、草原建设396万亩、水土流失治理180万亩。加强生态资源保护力度，实施灌木林平茬工程，推进自然保护区开发建设活动清理整治。严格执行禁牧、休牧、划区轮牧及草畜平衡制度，严厉打击非法开垦、破坏野生动植物资源等行为，切实巩固生态建设成果。

更严措施治理环境。认真贯彻落实《鄂尔多斯市环境保护条例》，保护好鄂尔多斯的蓝天净土绿地。实施工业污染全面达标排放工程，抓好煤化工和焦化行业脱硫升级改造、燃煤电厂超低排放改造等重点工作，确保空气质量优良率保持在85%以上。加强重点流域水污染和农村牧区环境综合整治。推进矿区地质环境治理和露天煤矿复垦绿化。持续提升环保监管能力，建成环境数据资源中心及生态环境监测监管系统。强化重点环境问题集中整治，加快棋盘井蒙西地区环境综合治理，切实抓好中央环保督察反馈问题整改落实。

更高水平利用资源。加强生态文明制度建设，健全生态保护补偿机制。开展环境污染第三方治理，发展生态环保产业。严格落实节能减排约束性指标，全面推进企业循环式生产、产业循环式组合、园区循环式改造，力争工业固废综合利用率达到80%以上。实施城市污水处理厂提标改造工程，再生水利用率达到60%以上。大力发展公共交通，引导居民低碳绿色出行。

（六）持续保障和改善民生，共享社会发展新成果。

巩固脱贫攻坚成果。坚持力度不减、措施不变、投入不降，聚焦脱贫人口和市低保线下贫困人口，继续落实好“六个精准”和“五个一批”要求，因户因人精准施策，“扶上马、再送一程”，确保脱贫群众不返贫，力争市低保线下贫困人口年收入增长20%以上。设立产业扶贫基金，全面实施产业扶贫项目，扶持脱贫人口发家致富，提高贫困户自我发展能力。

推动充分就业创业。深入实施“创业鄂尔多斯”行动，完善创业扶持政策和激励机制，加强创业创新平台建设，创业带动就业1万人以上。实施就业服务工程，积极开发就业岗位，加大对高校毕业生、返乡农民工、化解过剩产能下岗职工等重点群体的就业帮扶力度。全年新增城镇就业2.9万人，城镇登记失业率控制在3.1%以内。

提高社会保障水平。深化收入分配制度改革，建立机关事业单位基本工资正常增长机制，全面落实最低工资标准，切实增加城乡居民收入。建成全民参保数据库，扩大社会保险覆盖范围。城乡低保标准再提高6%，农村牧区特困人员集中和分散供养标准分别提高到每人每年1万元和6852元。完善城乡医疗救助和临时救助制度，促进社会福利、妇女儿童、残疾人事业健康发展，大力发展红十字等慈善公益事业。

办好人民满意教育。不断改善办学条件，新建、改扩建中小学、幼儿园26所，推进寄宿制学校生活设施标准化，完成市三中新校区整体搬迁。发展普惠性学前教育，提升基础教育质量，大力发展高等教育。深化校企合作，推进职业教育改革发展。加快教育信息化建设，实现城域网教育教学全覆盖。加强师资队伍建设，实施“名师梯队工程”。进一步改善农村牧区教师生活条件。

建设健康鄂尔多斯。深化公立医院改革，建立现代医院管理制度，完善基本药物制度，健全药品供应保障体系。建立全市统一的健康档案数据库，推进同级医疗机构检查检验结果互认，逐步实现异地住院医疗费用直接结算。改善群众就医环境，开工建设市儿童医院项目，确保市中医院新住院大楼投入使用。完善计划生育服务管理。加快蒙中医药事业发展，建设一批知名蒙中医药馆。

繁荣发展文体事业。持续推进“十个一”文化工程，推出一批文艺精品，开展好文化下乡、文化进社区活动。大力发展民族文化，做好文化遗产保护和传承工作。弘扬红色文化，确保鄂前旗“1+6”红色培训教育基地建成开放。扎实推进足球运动改革发展，建成自治区级足球训练基地。完善基本公共体育服务体系，引导全民重视健康、主动参与健身。

（七）创新社会治理方式，构筑平安稳定新优势。

不断提高安全生产水平。“天大地大，安全最大”。始终保持安全生产高压态势，严格落实安全生产责任制，督促企业落实主体责任，深入开展安全隐患明察暗访、专家巡查和第三方监管，坚决消除安全隐患。提升安全监管信息化水平，建成国内领先的公共安全信息化指挥调度平台，实现市内重点企业、重点部位、重大危险源实时监控，坚决杜绝重特大安全事故发生。

积极创建食品安全城市。加强检验检测能力建设，健全完善市、旗、苏木乡镇三级信息化综合监管平台，构建从源头到消费全过程监管格局。推进专项整治和综合治理，严厉打击食品药品安全违法犯罪行为，争创国家食品安全试点城市。

切实维护社会大局稳定。深化“七五”普法，落实“谁执法、谁普法”责任制。完善公共法律服务体系。深入开展矛盾纠纷排查化解，妥善解决群众反映的突出问题。强化应急预案管理和演练，提高突发事件防范应对能力。完善社会治安防控体系，建设平安鄂尔多斯。认真落实党的民族宗教政策，全面创建全国民族团结进步示范市。巩固双拥共建成果，促进军民深度融合发展。

（八）加强政府自身建设，营造干事创业新环境。

全力建设法治政府。严格按照法定权限和程序履行政府职能，坚决执行市人大及其常委会的决议和决定，坚持重大事项报告制度，组织实施政府协商计划，认真办理议案建议和提案。落实好市人大常委会立法工作计划。深化行政执法体制改革，全面推进综合执法。完善社会监督和舆论监督机制，做好政务公开工作，确保权力在法治轨道上规范运行。

全力建设效能政府。大力推进简政放权，推行互联网+政务服务，全面提升服务效率。强化执行力建设，实行政府目标管理，建立部门信息共享和协同配合机制。强化行政问责和效能监察，严肃整治不作为、慢作为和乱作为等行为。进一步精简会议和文件，办有效的事，开务实的会，发管用的文。探索建立合理的容错机制，鼓励干事，支持创新，最大限度激发广大干部干事创业热情。

全力建设责任政府。深入开展纠风行动和专项治理，坚决打击损害群众利益的不正之风和突出问题。大力推进政务诚信建设，认真履行政府承诺。严格执行“三重一大”制度，发挥市决策咨询委员会作用，开展重大行政决策第三方评估。厉行勤俭节约，反对铺张浪费，严控“三公”经费支出，集中更多财力用于民生改善、帮扶困难群众。

全力建设廉洁政府。自觉执行《准则》和《条例》，把纪律和规矩挺在前面。严格落实党风廉政建设主体责任和“一岗双责”，切实加强资源配置、土地出让、工程建设等重点领域监管，对各类违法违纪案件始终做到零容忍、严惩治。加大审计、监察力度，对公共资金、国有资产、国有资源和领导干部履行经济责任情况实行审计全覆盖。坚持用制度管权、管事、管人，切实做到干部清正、政府清廉、政治清明。

今年，我们将迎来自治区成立70周年大庆，这是全市人民政治生活中的一件大事，我们将以迎庆为契机，多为老百姓办实事好事，努力增加百姓福祉，进一步增强全市各族人民的荣誉感和使命感，激发起同心协力建设美丽家园的强大动力。同时，今年联合国防治荒漠化公约第十三次缔约方大会将在我市举行，这是国家“一带一路”战略的重要组成部分，我们将举全市之力承办好这次会议，讲好中国故事，讲好内蒙古故事，讲好鄂尔多斯故事，为党的十九大胜利召开创造良好的国际外交环境。

各位代表！做好今年工作，任务繁重，责任重大。让我们在中央、自治区和市委的坚强领导下，坚定信心，团结奋斗，开拓创新，一往无前，奋力开创各项工作新局面，为把鄂尔多斯打造成为祖国北疆亮丽风景线上更加璀璨的明珠而努力奋斗！
